# Cyclosporine as Monotherapy for Psoriasis in the Setting of Chronic HCV Infection: A Forgotten Therapeutical Option

**DOI:** 10.5812/hepatmon.6057

**Published:** 2012-05-30

**Authors:** Alexandra Maria Giovanna Brunasso, Paolo Michetti, Laura Fancelli, Cesare Massone

**Affiliations:** 1 Department of Dermatology, Galliera Hospital, Genoa, Italy; 2 Department of Environmental Dermatology and Venereology, Medical University of Grazp, Graz, Austria; 3 Department of Gastroenterology, Galliera Hospital, Genoa, Italy; 4 Department of Dermatology, Medical University of Florence, Florence, Italy; 5 Department of Dermatology, Medical University of Graz, Graz, Austria

**Keywords:** Psoriasis, Cyclosporine, Infection

## Abstract

**Background:**

Treatment of psoriasis in the setting of chronic hepatitis C virus (HCV) infection is difficult, because standard therapies like methotrexate are associated with increased hepatic toxicity. Due to the HCV suppressive effect. Cyclosporine may represent a valid systemic alternative for psoriatic-HCV patients.

**Objectives:**

In this study, we report the successful usage of intermittent cycles of cyclosporine in the setting of chronic HCV infection and we try to call the attention once again in a very effective and forgotten therapeutic option for severe chronic plaque psoriasis.

**Observation:**

We describe a 48 years - old patient who has a 20 year history of severe chronic plaque psoriasis and HCV infection (aminotransferase levels are three times normal; HCV genotype 2a-2c and HCV-RNA titer of 2.050.000 UI-ml). Five courses (range of duration of three to six months) of oral cyclosporine (5 mg/kg/day) were followed during a 38 month period. The viral load and the transaminases’ levels diminished during the 38 months of intermittent cyclosporine therapy to the lowest level measured at 36th month. The good psoriatic response was associated to a slight improvement of the liver condition, even though the HCV-RNA was reduced by less than 1 log10 without normalization of aminotransferase’ levels.

**Conclusion:**

The reduced liver toxicity, the potential anti-HCV properties and the well-known systemic anti-inflammatory effect, make cyclosporine a good alternative for recalcitrant psoriatic patients with HCV-liver disease

## 1. Background

Treatment of psoriasis in the setting of chronic hepatitis C virus (HCV) infection is demanding, because standard treatments like methotrexate are associated with increased hepatical toxicity [1]. Due to the HCV suppressive effect, cyclosporine may represent a valid systemic alternative for psoriatic-HCV patients [2]. Unfortunately in the last couple of years, only biological therapies such as anti-TNF- α agents have been considered as valid options for such patients, not considering the high costs related to the continuous use of these agents [1].

## 2. Objectives

Nowadays Cyclosporine is one of the most effective systemic therapies for chronic plaque psoriasis, and it is considered as second-line treatment after phototherapy for high needed psoriasis patients where no contraindications are present. Unfortunately the scarce literature evidence regarding the use of cyclosporine in the setting of chronic HCV infection and the increasing enthusiasm on biological therapies and mainly anti-TNF-α agents, have been made that recently published guidelines proposed the use of cyclosporine only as third line-therapy after the use of adalimumab, etanercept and/or infliximab. This report describes the successful usage of intermittent cycles of cyclosporine in the setting of chronic HCV infection.

## 3. Case Report

**Figure 1:**
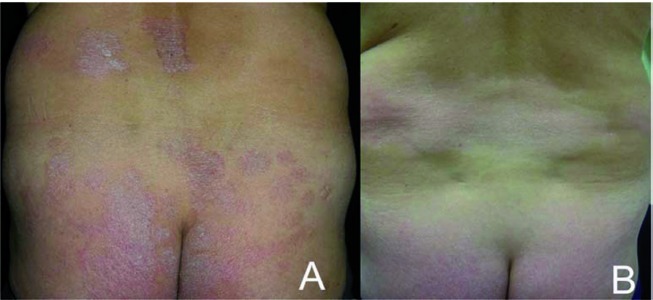
A: Erythematous-Squamous Lesions Involving The Back (Day 0; PASI: 19.8); B: Clinical Improvement Following Cyclosporine Treatment (38th month; PASI: 4.5)

**Figure 2:**
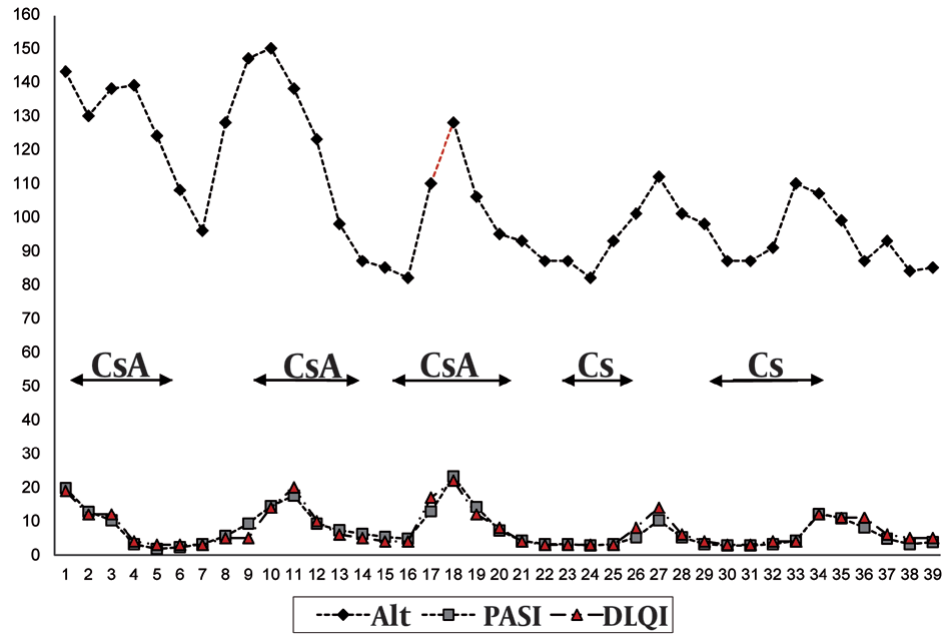
Psoriasis Severity Evolution (PASI and DLQI), Alanine Aminotransferase Levels and HCV-RNA During 5 Courses of Cyclosporine. HCV-RNA: measured by the quantitative-chain-reaction assay, detection limit, 50 IU/mL, units: X 104 IU/mL; Alt: alanine aminotransferase, unit: U/L; CsA: Cyclosporine; PASI: Psoriasis Area and Severity Index; DLQI: Dermatology Life Quality Index.

A 48 years-old white man was followed for a 20-years history of severe chronic plaque psoriasis. In 1990 HCV infection was demonstrated with raised liver enzymes and ongoing viral replication. The patient presented to us with persistently elevated aminotransferase levels (ALT; three times normal), HCV infection with genotype 2a-2c (Simmonds Classification) and HCV-RNA titer (measured by the quantitative-chain-reaction assay, detection limit, 50 IU/mL) of 2,050,000 IU/mL [3]. A liver biopsy showed moderate to severe fibrosis with moderate to severe portal, per-portal and globular mononuclear infiltrate (metavir A3, F3) [1]. The patient denied his consensus for interferon IFN-α/ribavirin therapy because of the possible worsening of psoriasis during IFN therapy. In the past, the patient received numerous topical courses of corticosteroids, vitamin D derivates, tar derivates and salicylic acid with partial improvement. Ultraviolet B phototherapy was introduced for 4 months with only slight response. At baseline, the patient presented with a Psoriasis Area and Severity Index (PASI) of 19. 8 (Figure 1A), a Body Surface Area of 19, with a severe palmo-plantar involvement that impaired his quality of life (QOL), measured by the Dermatology Life Quality Index (DLQI) questionnaire, with a score of 19 (range 0 to 30, being 30 the worse QOL). Because of the severity, extension and QOL impairment, systemic therapy was essential and cyclosporine was chosen in the HCV setting, considering the contraindication for methotrexate and the scarce response after two months of acitretin. Cyclosporine was introduced at 5 mg/kg/day with progressive tapering of the dose by 50 mg/weekly after PASI-90 achievement (90 % of PASI improvement; Figure 1B). During cyclosporine therapy, liver function tests (transaminases and gamma glutamil transferase) were measured every and HCV viral load was measured every six months using the same quantitative polymerase-chain reaction assay (detection limit, 50 IU/mL). A total of five courses (duration range: three - six months) of cyclosporine (5 mg/kg/day) were followed by the patient during a 38-month period. The viral load diminished during the 38 months of intermittent cyclosporine therapy to the lowest level measured at month-36 (6.5 x 105 IU/mL). The transaminases levels diminished during the whole therapy, with a 42 % of improvement from baseline to 38th month (Figure 2). Blood pressure, triglycerides, total or LDL-cholesterol levels and renal function remained on the normal range during the whole cyclosporine intake period.

## 4. Discussion

Treatment of recalcitrant psoriasis in the setting of chronic HCV infection is difficult and guidelines from the medical board of the national psoriasis foundation recommend the use of cyclosporine only as third-line therapy after a second-line therapy with etanercept, adalimumab, infliximab and/or etanercept [4][5][6]. Anti-HCV therapy (IFN-α) may also aggravate psoriasis [7][8]. Recently, a systematic review of the literature showed that the safety profile of anti-TNF-α agents such as adalimumab, etanercept and infliximab, seems to be acceptable, but the absence of long-term and large, controlled clinical trials prevent from making a definitive statement on the safety of anti-TNF-α therapies in the setting of chronic HCV infection [1]. Our case report demonstrates long-term safety (38 months of follow-up) and good clinical response after intermittent treatment with cyclosporine. Considering the cost/ benefit-risk ratio, cyclosporine remains in the majority of clinical guidelines as a second line-option for chronic plaque psoriasis and should always be used before the introduction of biological therapies when no contraindications are present [9]. In the setting of HCV infection, we agree with Di Lernia et al. that cyclosporine should remain a second-line therapy for psoriasis and that should be used before anti-TNF-α agents because of the cost-benefit ratio and the safety profile, considering the same level of evidence (case series) when compared to anti-TNF-α agents [10]. Cyclosporine has potential anti-HCV effects which are independent of its immunosuppressive functions [2]. The most likely mechanism of action against HCV is the blockage of the PPIase (peptidyl - prolyl cis-trans isomerize) activities of cyclophilins, inhibiting molecular chaperones that accelerate the slow steps of correct post-translational folding of some viral proteins [11]. The sustained virological response is higher in patients carrying genotype 1 HCV in comparison to genotype 2. HCV genotype 1b is highly sensitive to cyclosporine, in contrast with the slight response of fulminant-type genotype 2a replicon (JFH1), even if high cyclosporine doses can suppress the replication of this strain in vitro [12]. The scarce cyclosporine sensitivity has not been confirmed for the chronic-type genotype 2a, because replicon does not yet exist [12]. Cyclophilin A is the principal mediator of cyclosporine resistance in vitro; removal of cyclophylin A from resistant replicons cells eliminates resistance [13]. In this case, the HCV-RNA was reduced but less than 1 log10, associated to a 42 % improvement of transaminases (without normalization of aminotransferase levels), which cannot be considered as a significant virological response [6]. Considering the HCV genotype 2a-2c of our patient, the non-significant virological response to cyclosporine is consistent with literature data [12]. The decrease of systemic inflammation can be related to the transaminase improvement as an indirect effect of cyclosporine, possibly unrelated to the antiviral properties of the molecule. The reduced liver toxicity and the HCV suppressive properties make cyclosporine an alternative for recalcitrant psoriatic patients with HCV-liver disease even if the anti-virological effects are not well quantified, as reported in one case by Imafuku and co-workers [8]. Patients with HCV genotypes 1, 3 and 4 might benefit in a larger degree from the suppressor HCV properties of the molecule. One trial had demonstrated the beneficial effect of combined IFN and cyclosporine in liver transplant non-psoriatic patients with HCV recurrence, but no clinical trials have been performed so far to demonstrate the anti-HCV effects of cyclosporine in mono-therapy [14][15]. Further studies are needed in order to demonstrate whether the anti-HCV effect of cyclosporine is clinically and virologically relevant. An estimated 2.7 % HCV prevalence in an Italian cohort of 3577 serum samples in association to the high psoriasis prevalence (1-3 %), makes the co-mobility between these two conditions an everyday practice dilemma between: patients afraid of IFN-α psoriasis rebound, dermatologist discouraging anti-HCV treatment and gastroenterologist ignoring the high psoriasis morbidity related to QOL impairment [8][16]. At the end of this triangle, very often, the patient found him or either treated for the liver condition (accompanied with psoriasis worsening) or insufficiently treated for the cutaneous disease because of the huge concerns about drug safety from dermatologists. We encourage a multi-disciplinary approach in order to treat both skin and liver conditions as effective as possible for every single case.
